# Pakistan Ranks Third Globally With the Most Unvaccinated Children: Is the Impact of Parental Perception and Attitude on Immunization an Essential Contributing Factor to an Unsuccessful Vaccination Coverage?

**DOI:** 10.7759/cureus.19751

**Published:** 2021-11-19

**Authors:** Rabail Saeed, Irtiza Hashmi

**Affiliations:** 1 Community Health Sciences, Ziauddin University, Karachi, PAK; 2 Biochemistry, Ziauddin University, Karachi, PAK

**Keywords:** public health, polio, polio eradication, pakistan, vaccination, immunization, health education, caregiver education, higher education, parents

## Abstract

Introduction

Having the third-highest burden of child mortality and ranking third globally for the most under-vaccinated children, Pakistan contains 15% of its population under the age of five, which accounts for 50% of the mortality rate in this country. Every year, almost three million children miss out on an entire course of the most readily available vaccines, leaving them vulnerable to life-threatening diseases. The Expanded Program of Immunization (EPI) was launched in 1978 to protect children from vaccine-preventable childhood diseases. It is the main program through which routine immunization is provided to the public. However, since its inception within Pakistan, it has encountered many problems, including a lack of parental awareness and education. Low literacy rate, socio-economic disparities, cultural and religious beliefs have made parents doubtful about vaccinations. This term is known as vaccine hesitancy. Belief in conspiracy theories has also led to reduced vaccination coverage in Pakistan.

Methods

A cross-sectional study was conducted on 300 parents with children under five, chosen from Karachi, Pakistan, through a convenience sampling technique. The data was collected via 300 self-administered or researcher administered questionnaires. Associations were evaluated using the chi-square test with the level of significance taken as *p* < 0.05 and Cramer's V to determine the strength of these associations. Data analysis was done using Statistical Package for the Social Sciences (SPSS) version 20.0.

Results

Strong associations were found between parental knowledge of immunization, vaccines, and willingness to get their children immunized. Associations were greater and significant in parents with a higher level of education compared to lower. However, lack of knowledge about certain essential vaccines like Pentavalent, Pneumococcal, and IPV was an important aspect to consider. Also, it was noted that their inability to access these free vaccines was due to their own firm beliefs. It was also about the lack of awareness and facilities for a better and beneficial program. 22.3% of parents said that administering multiple vaccines at a time would be harmful to their child, and 21.7% believed it would cause the disease it's supposed to prevent. However, the majority of the parents said they would strongly recommend others to get vaccinated. In this study, religions included and investigated mostly encouraged vaccination to their relatives (p value= 0.079). Occupation and Monthly income had little to no effect on the immunization regime of young children.

Conclusion

An impact of higher levels of education on the perceptions and attitudes of parents regarding the immunization of children was noted. Consequently, as religion, gender, occupation, and monthly income do not affect parents' perceptions regarding immunizations, the problem lies within their knowledge and understanding of basic medical science and easily communicable diseases. Oblivious to the consequences of contracting a lethal disease, it has developed a laid-back attitude amongst parents. Hence, awareness and education of parents regarding vaccine-preventable diseases by the healthcare system and the governing bodies can lead to a higher successful immunization rate.

## Introduction

In 1978, The Expanded Program of Immunization (EPI) was launched in Pakistan to protect children from fatal, vaccine-preventable childhood diseases. Preventing potentially dangerous childhood illnesses like Poliomyelitis, Tuberculosis, Diphtheria, Pertussis, Pneumonia, and Meningitis, these vaccines have proved to provide immunity worldwide. However, it ranks third globally for the most under-vaccinated children. Lagging, compared to globally standardized immunization targets, is still among the few remaining countries that still have poliovirus cases being reported [1]. According to WHO, 2 to 3 million deaths occur each year which can be prevented by immunization, of which approximately 1.5 million deaths occur in children less than five years of age [2,3]. Some essential vaccines covered by EPI are Bacille Calmette-Guérin (BCG), OPV, IPV, Pentavalent, Pneumococcal, DPT, Rotavirus, and Measles. 

In Pakistan, efforts for vaccination campaigns have faced many impediments, including vaccine hesitancy, religious misconceptions, and poor coverage. Parents are often doubtful about vaccinations causing them to delay or refuse readily available vaccines. This term is known as 'Vaccine hesitancy' [4]. According to a poll, 77% of parents expressed concerns regarding one or more childhood vaccines [5]. One of the reasons is that vaccinations have contraindications, and their misconceptions have often been reported as causes of unnecessary delay in vaccine administration [6]. In vaccinated individuals, vaccines were viewed as less efficient in disease prevention. They were often confused as to which illnesses the vaccines would defend against.

In contrast, unvaccinated individuals were more worried about unidentified, long-term vaccine side effects than with the illness it prevents. Many mothers thought that it was not always feasible to prevent disease [7]. However, studies have shown that parents with higher levels of education have significant knowledge and understanding about immunizations. The use of available vaccines has greatly decreased the cost of morbidity, death, and healthcare-associated with contracting an infectious disease. One of the main factors attaining successful vaccinations is parental beliefs about immunizations [8].

The purpose of this study is to evaluate whether parental perceptions have hindered Pakistan's effort to achieve its immunization targets. Furthermore, we also assess how education has dramatically influenced parental insight and awareness regarding immunization. Analyzing public knowledge of vaccine benefits and consequences of missing them, then bringing improvement and foreseeing solutions on how to tackle misconceptions remains an essential aspect of this study.

## Materials and methods

A total of 300 parents took part in this study, all having children less than five years of age, using the convenience sampling technique. The data was collected via 300 self-administered questionnaires. They consisted of multiple-choice and open-ended questions, which were written in English. However, the participants who were not fluent in English or had trouble understanding the questions were helped by researchers who translated the questions to the native language Urdu, word by word. Answers were recorded accordingly to minimize bias. Every question had five or more responses; for example, a question asking about the place of birth of an individual included tertiary care hospital, primary health clinic, home, or midwifery clinic as options. Thirty of the total three hundred questionnaires were tested for face validity in a pilot study, the results of which were consistent with our expectations. Personal data of participants was not noted nor required, and written consent was taken before recording any data. Variables like education, religion, gender, and monthly income hypothesized in previous literature were selected, and their association with parental beliefs and perceptions were recorded while maintaining anonymity. In addition, the age, gender, and ethnicity of the child and parent were also recorded. Associations were further examined to see the strength of these variables' impact on the young generation's parents. 

A cross-sectional survey designed to assess parents' knowledge about vaccinations and immunizations also included names of all the EPI vaccines and whether participants knew about them. Furthermore, participants were asked to choose from the listed reasons or otherwise state reasons for not vaccinating their children, skipping vaccines, and not following up on boosters. Reasons included unavailability of vaccines at the facility, long waiting times, and far-off health facilities. They were also questioned whether they thought their child needed a vaccine or knew what vaccination was. Questions like, 'Do you think the administration of multiple vaccines is harmful?' and, 'Do you think vaccination is important?' were asked to determine if parents had any experience or knowledge related to this subject. To make it easily comprehensible, options from 'Yes' or 'No' were given to them to choose from. 

The duration of the study was eight months, from January 2019 to September 2019, with data collected from Pediatric outpatient departments of three different tertiary care hospitals (Ziauddin Hospital Clifton, Ziauddin Hospital Keamari, and Ziauddin Hospital North Nazimabad), family fun and recreational centres in different areas, and amusement parks located across Karachi, Pakistan. Employees working for the government's vaccination program and NGOs were excluded from this study.

Data was entered into IBM Statistical Package for the Social Sciences 20.0, and frequencies were calculated using descriptive statistics. The chi-squared test was used to find associations of demographic variables with knowledge/attitudes of parents towards immunizations with p-value < 0.05 taken as significant, and Cramer's V test was used to measure the strength of the associations.

## Results

Out of 300 parents who participated in the study, most were females (86.3%), most of whom did not work outside their homes and were mainly housewives (68.8%). Level of education did not vary significantly among the participants; 24.3% had no educational background, 19% had completed their Matric/Ordinary/O level or High School, 13% had completed their Intermediate/ Advanced/A level or College, and 22.7% and 21% were Graduates and Post-Graduates, respectively.

**Figure 1:**
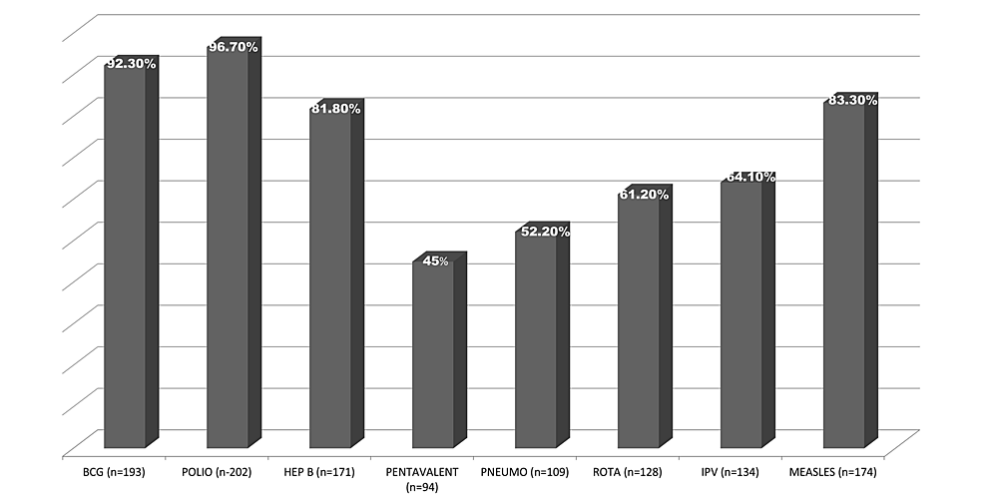
Percentage of people aware of Pakistan's Expanded Programme on Immunization (EPI) and its Vaccines. x-axis = Different EPI vaccines. 'n' represents the number of people out of 209 who are aware of the EPI and its vaccines y-axis = Percentage of people who are aware of these vaccines Pentavalent and Pneumococcal stand to be the least known vaccines whereas people seem to be more aware of the Polio vaccine.

**Figure 2:**
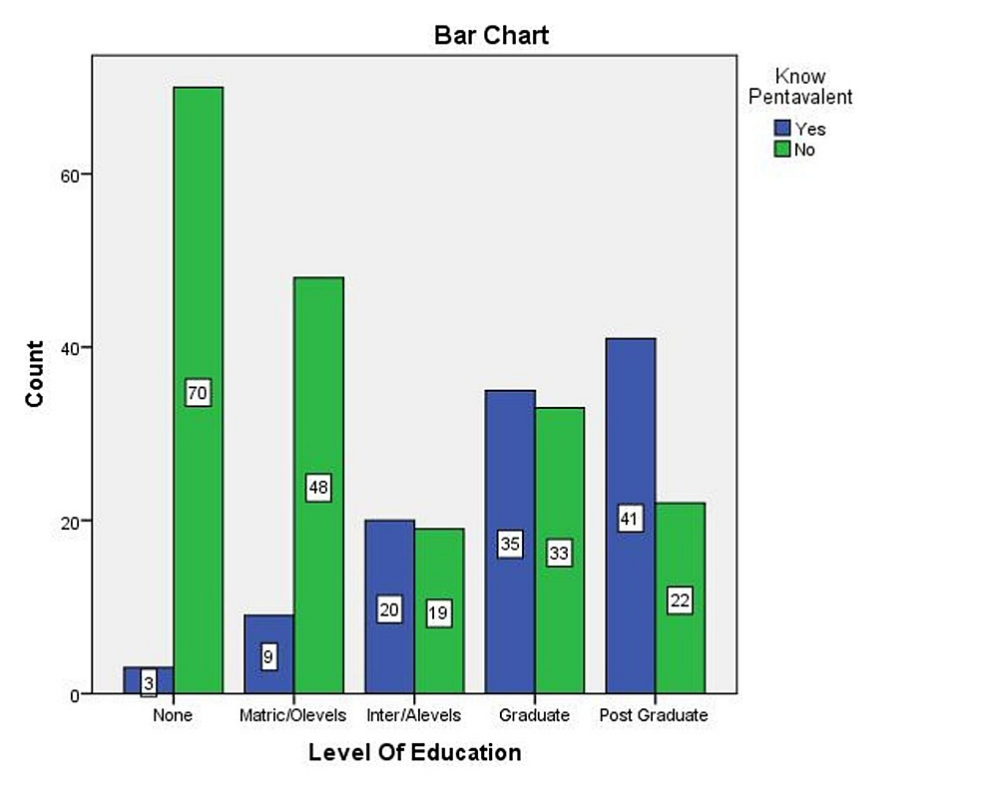
Number of people aware about Pentavalent Vaccine according to their level of Education (n=300) x-axis = Different levels of education parent's belonged to y-axis = Number of people aware of this vaccine with respect to their level of education

**Figure 3:**
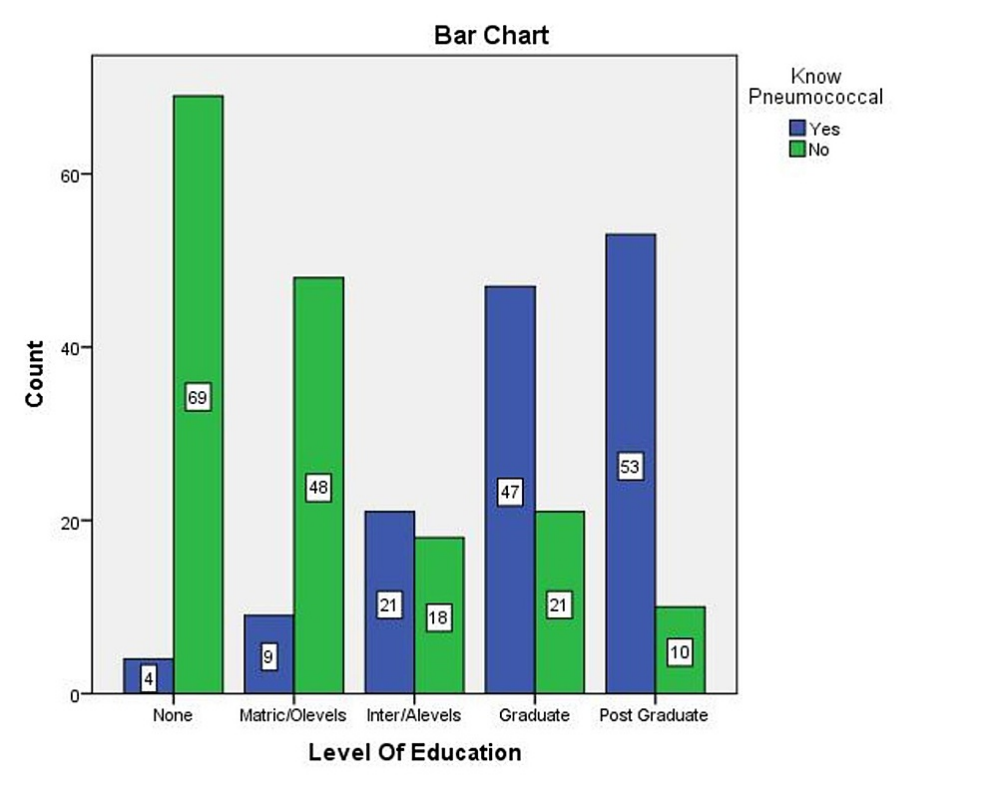
Number of people aware about the Pneumococcal vaccine according to their level of Education. (n=300) x-axis = Different levels of education the parent's belonged to y-axis = Number of people aware of this vaccine with respect to their level of education

69.7% of the parents said yes when asked if they knew about Pakistan's immunization program (EPI) and its vaccines, and 26.7% said no (Figure 1). Even though most of the participants claimed to know about EPI, most were not aware of the EPI vaccines or schedules, especially about the Pentavalent vaccine (45%) and the Pneumococcal vaccine (52.2%). When asked about these respective vaccines, numbers varied greatly amongst participants from different educational backgrounds (Figure 2, 3). Associations were significant between higher levels of education and knowledge about EPI vaccines amongst parents (Table 1).

**Table 1:** Association of the level of education with knowledge about EPI vaccines using the chi Square test and Cramer's V Associations were significant between higher levels of education and knowledge about EPI vaccines amongst parents. *p-value < 0.05 BCG- Bacillus Calmette Guerin; Hep B- Hepatitis B; IPV- Inactivated polio vaccine

Vaccines	p-value	Cramer's V
BCG	<0.001*	0.33
Poliovirus	0.000516*	0.258
Hep B	<0.001*	0.416
Pentavalent	<0.001*	0.505
Pneumo	<0.001*	0.638
Rotavirus	<0.001*	0.609
IPV	<0.001*	0.426
Measles	<0.001*	0.473

Various reasons stated by the majority of the participants who did not get their children vaccinated at birth included, child, being born at home (28.1%), they did not think that their child needs vaccination (18.6%), or that they were not aware what vaccination is (18.8%).

Forty-three out of the total participants did not follow up on the booster doses for their children. To inquire about the reason as to why that is, they were asked to choose from the following list provided, and the most common responses were recorded and tabulated (Table 2). 23.3% said that the healthcare facility did not inform them about the booster dose or forgot about it themselves (18.6%). There was, however, a significant association between parents' level of education and parents following up on boosters (p-value < .05, Cramer's V= 0.223). The majority of the parents who did not follow up on boosters had a low level of education; only 68% with no educational background followed up on boosters. Furthermore, most parents with a low monthly income did not follow up on boosters (p-value < .05).

**Table 2:** Common causes for parent refusal or skipping of vaccines. 91 out of 300 participants had skipped their child's vaccine; 43 out 300 participants did not follow up on boosters.

Common Reasons parents skipped a vaccine	Percentage	Frequency
Live Far Away From Health Care Facility	18.70%	17
Long Waiting Time	17.60%	16
No Vaccine Available At That Day	19.80%	18
No Information About The Day Of Vaccination	8.80%	8
Child Was Sick So Could Not Get Vaccine	9.90%	9
Child Fell Sick After First Dose	9.90%	9
Laziness	7.70%	7
Did Not Think It Was Necessary	0.70%	2
No Time To Go	0.70%	2
Vaccine Was Expensive	0.70%	2
Did Not Know About Vaccination	0.30%	1
Total	100%	91
Common Reasons parents did not follow-up on booster		
Was Not Informed By The Health Care Facility	23.30%	10
Forgot About It	18.60%	8
Lost The Vaccination Card And Could Not Recall Date For Follow up	9.30%	4
Socioeconomic Reasons	11.60%	5
Laziness	9.30%	4
Did Not Have Time To Go	7.00%	3
No Hospital Nearby	9.30%	4
Child Fell Sick	11.60%	5
Total	100%	43

Ninety-one of the total parents had skipped one or more vaccines included in the vaccine regime. This was all due to no vaccine being available on the day of vaccination (19.8%), living far away from the healthcare facility (18.7%), or having a very long waiting time at the facility (17.6%) (Table 2). The association between parents' Educational background and skipping vaccines was significant (p-value < .05, Cramer's V= 0.232). Only 20.6% of the parents with a higher level of education had skipped a vaccine, whereas 49.3% of parents with no educational background had skipped a vaccine for their child.

Sixty-seven parents out of the total said that administering multiple vaccines at a time would be harmful to their child, whereas 11.7% were sceptical and said they did not know (Table 3). However, this had no association with their education level (p-value= .293). 21.7% of the parents believed vaccines to cause the disease. It is supposed to prevent and significantly associated with their educational background (p-value = 0.017). However, 91% of the parents said they would recommend and advise their family and friends to immunize their children.

**Table 3:** Parental Perceptions Regarding Vaccines.

Variable	Response Recorded		
	Yes	No	Don't Know
Think Vaccine Is Important	265 (88.3%)	15 (5.0%)	20 (6.7%)
Think Vaccination Works	257 (85.7%)	12 (4.0%)	31 (10.3%)
Multiple Vaccines Are Harmful	67 (22.3%)	198 (66.0%)	35 (11.7%)
Think Vaccines Outweigh Benefits	44 (14.7%)	201 (67.0%)	55 (18.3%)
Think Vaccines Cause The Disease Its Supposed To Prevent	65 (21.7%)	184 (61.3%)	51 (17.0%)

There was also a significant association (p-value < .001, Cramer's V= 0.249) found between the level of education and parental perception of vaccination being important for their child. 95-100% of parents with higher education thought that vaccination was important, while only 74% of parents with no educational background thought vaccination to be important.

Parents' knowledge and perceptions of immunization were not associated with the number of children they had. There was an association between the number of children and parents who still have the vaccination cards of their children (p-value= .008). The majority of the parents with children more than four or five had lost their vaccination cards. 

Gender and religion did not significantly impact the knowledge and perceptions of parents regarding immunizations (Table 4). However, occupations relating to a higher level of education had a significant association with the attitudes and perceptions of parents. Parents with jobs of higher status had sufficient knowledge about EPI (p-value = .018), they did not think that administration of multiple vaccines is harmful (p-value < .0001), did not think vaccines cause the disease it's supposed to prevent (p-value= .013), and also think that vaccination is important for their child (p-value < .001). Monthly income had little to no effect on the immunization regime of young children.

**Table 4:** Associations of Parental Perception to different variables assessed like occupation, monthly income, gender of the child, religion and number of children, using the chi-square test and Cramer's V *p-value < 0.05 Strong associations were seen in occupations of higher status and parental perception attitude regarding vaccination.

Variable		p-value	Cramer's V
	All children vaccinated	<0.01*	0.485
Occupation	Followed up on boosters	<0.01*	0.396
	Think vaccination is important	<0.01*	0.372
	All children vaccinated	0.191	0.123
Monthly income	Followed up on boosters	<0.01	0.204
	Think vaccination is important	0.059	0.145
	All children vaccinated	0.021	0.158
Gender	Followed up on boosters	0.057	0.145
	Think vaccination is important	0.003	0.181
	All children vaccinated	0.367	0.085
Religion	Followed on boosters	0.063	0.124
	Think vaccination is important	0.839	0.049
	All children vaccinated	0.252	0.169
Number of children	Followed up on boosters	0.024	0.212
	Think vaccination is important	0.036	0.204

## Discussion

According to this study, higher education did indeed influence parents' awareness, comprehension, and attitudes towards immunizations. It was found that parents with higher-status jobs were more receptive to the vaccine policies and more willing to get all their children fully vaccinated. However, gaps in their knowledge hinder their ability to judge its importance as the parents are not aware of the predominantly fatal diseases in their region and the risk of being exposed to them by their child. Efforts to increase vaccination coverage should consider the factors leading to children's inadequate vaccination status [9].

Children in Pakistan receive vaccines at fixed primary health centres managed through the EPI. Specific outreach initiatives, such as National Immunization Days, are also implemented. Factors associated with increased immunization dropout rates include parental challenges in obtaining healthcare services and insufficient monitoring of healthcare workers in healthcare facilities [10]. Immunization services, parental awareness, and their behaviour were the main reasons for under-vaccination. The reasons most often cited also included access to care, quality of services, false contraindications, practical knowledge of vaccination by parents, fear of side effects, contradictory interests, and parental values [11]. In this study and as stated in (Table 2), we found that the majority of parents who had missed opportunities to get their children vaccinated were mostly because the child was born at home (28.1%), the health care facility did not inform them about the next vaccination (23.3%), no vaccine or personnel were available at the facility at the day of vaccination (19.8%), they were living far away from health care facilities (18.7%), they did not think that their child needs vaccination (18.6%), or that they were not aware what vaccination is (18.8%). Some of the parents even stated that it was because of their carelessness, reflecting poor judgment on their part as these parents are not aware of the damages or the consequences of not getting their children immunized. Parents who delayed and denied vaccine doses were more likely to have concerns about vaccine safety, so they perceived fewer vaccine-related benefits [12]. A study among 12-24-month-old children showed that 50% of them were fully immunized, 31.3% were partially immunized, and 18.7% were not immunized at all [13]. However, according to previous studies, there is limited data on the effectiveness of interventions to tackle the growing risk of parental denial of vaccines [14]. 

Information can play a key role in determining an individual's health actions. Lack of access for mothers to information raises their children's risk of inadequate immunization. Public awareness of immunization in Pakistan is generally very low, particularly among mothers from poor socio-economic backgrounds [15]. One of the essential findings in this study was that parents with a lower educational background lacked knowledge about EPI and its vaccines compared to parents with higher educational backgrounds. Pentavalent, Pneumococcal, and IPV vaccines were seen to be unheard of by most of the parents [Figure 1]. However, parents were well aware of the other vaccines like Oral Polio, Measles, Hepatitis B, and BCG. These are more widely reported by social media and are most frequently discussed. This study also found that parents did not clearly understand the importance of immunizations due to poor medical education. Only 74% thought vaccination important, and about 49.3% of them had skipped a vaccine. 29% of them did not follow up on boosters altogether. The poor literacy rate among these parents shows their casual attitude regarding their child's health. Furthermore, health education should also notify health workers about records and familial concerns about vaccination and keeping vaccination cards [16].

In addition to cost, low vaccination levels can be correlated with many other different causes. However, only a few studies discuss socio-economic inequality as a possible obstacle to achieving higher vaccination rates [17]. Likewise proved by this study, the majority of parents who had a low monthly income did not follow up on boosters (p-value < .001) [Table 4].

The knowledge of vaccine-preventable diseases among adults ranges from 63.4% to 94.0% [18]. In this study, 69.7% of parents claimed to know about the vaccination program of Pakistan. However, when probed about the vaccines individually, most parents were unaware of them or had never heard of them, especially Pentavalent and Pneumococcal [Figure 1]. A reason why that may be is majorly related to their educational backgrounds and their knowledge about immunizations. In one study, just 26.6% of parents knew that the varicella vaccine was accessible. The number of doses and awareness was significantly greater among university degree holders, those who had obtained vaccination details from a health care provider, and those who had vaccinated their child [19]. Vaccine information is most commonly provided to parents by health care professionals. However, families with exempt children were more likely to check with other sources for information on vaccines. Parents of vaccinated children were more likely to consider medical and public health sources as good or excellent for vaccination than parents of exempted children [20].

During the past 40 years, vaccines have proven to be one of the most effective weapons against child mortality [21]. In another study, a steady decrease in the average annual prevalence of antenatal HBsAg carriage was observed with a reliably significant decline in the three cohorts for 2007-2008 and 2010-2015. The study showed a significant reduction in the prevalence of HBsAg carriage in the standardized hepatitis B infancy [22]. With the emergence of new vaccines, immunizations can further reduce childhood mortality and provide broader health and economic advantages [21]. To minimize missed opportunities, efforts to improve the immunization program should include training vaccination staff to foster initiative. In the US, most states and the Columbia District have laws that require children to be vaccinated to enter public schools [23]. Public immunization campaigns have created major impacts on childhood diseases such as measles and mumps [24], including the potential of regional spread disruption, by improving permanent herd immunity and indirect security for the non-immunized [25].

## Conclusions

This study shows that, despite parents having a highly educated background, a majority had inadequate information or beliefs about immunizations. This is maybe secondary to the gaps in their knowledge and perceptions. These gaps seem to impact parents negatively and impair their complete understanding of the importance of vaccination. Healthcare providers need to educate adults about vaccine-preventable diseases, their exposure, the risks, and prevention. To meet standard immunization targets, public awareness is necessary to understand the importance of vaccination and increase its coverage. The health authorities should take the necessary steps to carry out mass awareness campaigns. The availability of vaccines and far-off healthcare units proved to be the greatest hindrance for parents to follow up on boosters. Free vaccination camps should be set up in remote areas to facilitate the underprivileged, and identification of children with missed usual doses are required to minimize incomplete vaccination regimes and boost immunization rates in Pakistan. 
